# Anesthetics in status epilepticus: does one size fits it all?—A scoping review on titration goals, timing, and patient selection

**DOI:** 10.3389/fneur.2026.1805775

**Published:** 2026-04-13

**Authors:** Urs Fisch, Pia De Stefano, Sira M. Baumann, Stephan Rüegg, Raoul Sutter

**Affiliations:** 1 Department of Neurology, University Hospital Basel, Basel, Switzerland; 2 Neuro-Intensive Care Unit, Department of Intensive Care, University Hospital of Geneva, Geneva, Switzerland; 3 Department of Clinical Neuroscience, University of Geneva, Geneva, Switzerland; 4 Intensive Care Unit, Department of Acute Medicine, University Hospital Basel, Basel, Switzerland; 5 Faculty of Medicine, University Basel, Basel, Switzerland

**Keywords:** burst suppression, EEG monitoring, electroencephalography, outcome, prognosis, refractory status epilepticus, seizure cessation, treatment

## Abstract

Status epilepticus (SE) is a neurological emergency with significant morbidity and mortality. Refractory status epilepticus (RSE) occurs in 20–30% of cases and may require treatment escalation to continuously administered intravenous anesthetic drugs (CIVAD). Despite widespread use, fundamental questions remain unresolved regarding optimal anesthetic management. This narrative scoping review examines CIVAD use in non-anoxic adult SE, focusing on three critical questions: What is the optimal titration goal? When should CIVADs be initiated and discontinued? Which patients benefit from CIVAD therapy? Regarding titration targets, current evidence does not support burst suppression as superior to seizure cessation for most RSE patients. Regarding timing, studies examining first- or second-line CIVAD administration have shown conflicting results, although a delayed initiation when used as a third-line treatment is associated with worse outcomes. Patient selection remains particularly challenging, as limited evidence supports aggressive CIVAD use in refractory nonconvulsive SE (NCSE) without coma or focal motor SE, whereas individualized approaches appear necessary for high-risk populations, such as NCSE with coma. The available evidence consists primarily of observational studies with inherent limitations. CIVAD therapy requires individualized decision-making based on SE type, patient characteristics, and etiology rather than standardized protocols. Future research should focus on prospective studies, advanced EEG analytics, and identification of robust biomarkers to enable precision medicine approaches in RSE management.

## Introduction

Status epilepticus (SE) represents a neurological emergency with significant morbidity and mortality that frequently requires intensive care management (1). Persistence of SE despite the administration of benzodiazepines and second-line antiseizure medications (ASM) defines refractory status epilepticus (RSE), occurring in 20–30% of SE cases (2–4). International guidelines recommend induction of a deep coma by continuously administered intravenous anesthetic drugs (CIVAD) as third-line therapy, typically propofol, midazolam, or barbiturates, for 24–48 h, targeting either electroencephalographic (EEG) seizure cessation, burst suppression, or complete suppression for 24–48 h (5–7).

The rationale for CIVAD use in RSE stems from the pathophysiology of prolonged seizure activity. Neurobiological changes during SE include alterations in gamma-aminobutyric acid (GABA) receptor configuration and trafficking, chloride-dependent depolarization, and N-methyl-d-aspartate (NMDA) receptor upregulation, leading to benzodiazepine-pharmacoresistance, rendering seizures progressively more difficult to terminate (8). CIVADs provide potent GABAergic enhancement and, in the case of Ketamine, NMDA receptor antagonism, potentially overcoming these resistance mechanisms through supraphysiologic potentiation of synaptic and extrasynaptic GABA receptors (9), or NMDA receptor blockade, respectively.

Several studies have demonstrated an independent association between CIVAD use and adverse outcomes in RSE, calling for heightened awareness of appropriate patient selection (10–13). These reports highlight concerns regarding potential iatrogenic harm linked to high doses and prolonged duration of anesthetic treatment (14). On the other hand, given that SE becomes increasingly refractory over time, early aggressive intervention has been proposed as a strategy to prevent progression to pharmacoresistance (15). This has led to considerations that ultra-early administration of CIVAD could prevent SE from becoming refractory when applied before conventional second-line therapies fail and could thereby also shorten the duration of anesthesia itself (14).

Recent publications have challenged traditional approaches regarding the use of anesthetics in RSE, prompting re-evaluation of long-standing practice patterns. In this scoping review, we critically examine current evidence on guideline-recommended third-line CIVAD use in adult SE with an emphasis on non-anoxic etiologies, addressing three fundamental questions: (1) What is the optimal CIVAD titration goal? (2) When should CIVAD be initiated and stopped? (3) In which SE patients is CIVAD most effective?

As this work is structured as a narrative scoping review, we conducted a structured literature search in PubMed focusing on publications addressing CIVAD use in adult status epilepticus, with emphasis on the past two decades, while also including seminal earlier studies.

## How deep should we go? The question of CIVAD titration goal

Depending on the depth of anesthesia, the EEG background changes from continuous to discontinuous to burst suppression to complete suppression. Burst suppression is a characteristic EEG pattern defined by alternating epochs of serial high-voltage discharges (bursts) and electrical suppression or attenuation (16). Historically, burst suppression has been widely advocated as the treatment target for RSE (17–20). A multinational survey among Austria, Germany, and Switzerland from 2003 revealed that up to 70% of respondents considered burst suppression the suitable titration target for RSE treatment (21). In a comparable 2025 survey conducted in the same countries, approximately 35% of respondents continued to favor burst suppression, indicating that this paradigm had become less stringent but is still prevalent (22).

The first edition of the American Clinical Neurophysiology Society’s (ACNS) Standardized Terminology Critical Care EEG Terminology in 2012 defined burst suppression as requiring ≥50% suppression, albeit this is a pragmatic but arbitrary threshold (23). Prior to this standardization, many studies did not specify the suppression ratio, making historical comparisons problematic (18).

Burst suppression is not always achievable and, when reached, may not be stable over time, with remarkable inter-patient and intra-patient variability in suppression proportions despite constant CIVAD administration (24, 25). To add another level of complexity, intracranial recordings have demonstrated that burst suppression is, in fact, a regional cortical phenomenon despite its global appearance on scalp EEG (26). Thus, burst suppression, despite its seemingly well-defined appearance on scalp EEG, may be difficult to reliably establish and maintain in clinical practice. In addition, Ketamine on its own does not typically elicit a burst suppression, but rather alternating epochs of gamma oscillations and delta waves (gamma bursts) (27).

Practical implementation varies substantially across intensive care settings. Continuous full-montage EEG with expert real-time interpretation is not universally available, particularly outside tertiary centers or during off-hours. In such contexts, burst suppression may persist as a favored target partly because it represents a visually recognizable EEG pattern, even for non-specialists and may be approximated using reduced electrode montages or amplitude-integrated EEG (28). Therefore, EEG target selection must account for available monitoring infrastructure.

A systematic review focused on the initial CIVAD selection among 66 studies until 2023, including 1,637 patients with RSE (29). When seizure etiology was taken into account, no significant differences in mortality were observed between midazolam, propofol, and barbiturates. However, these agents differed in their efficacy and adverse effect profiles. Barbiturates were associated with fewer treatment failures than midazolam or propofol (10% vs. 23% vs. 42%), but at the cost of substantially higher rates of hypotension (82% vs. 44% vs. 40%). A more granular subset analysis suggested higher mortality when seizure suppression, rather than burst suppression, was used as the treatment target. This finding, however, was based on limited data from 22 patients from 3 publications compared with 98 patients from 12 publications targeting burst suppression, severely constraining interpretation. Ketamine was minimally represented in the review, with only 10 patients across 3 eligible studies, precluding meaningful conclusions regarding its effectiveness. Notably, the review also observed a temporal trend toward decreasing mortality over time, although this association lost statistical significance after adjustment for clustering effects (*p* = 0.07).

In a retrospective single-center study published shortly after the aforementioned systematic review, we examined the frequency of induced EEG burst suppression during CIVAD therapy and its association with clinical outcomes in adult patients with RSE over a 9-year period (30). Using semiquantitative EEG analysis, burst suppression was categorized as incomplete (≥20 and <50% suppression) or complete (50–99% suppression), in accordance with the current ACNS definition (16). Among 102 patients without cerebral anoxia, complete burst suppression was achieved in 21%, with a median duration of 51 h, and was associated with more frequent hypotension requiring vasopressors but not infections. Multivariable analyses demonstrated no association between burst suppression and persistent seizure termination, in-hospital survival, or return to premorbid neurological function. These findings were consistent when considering burst suppression duration and when analyzing complete and incomplete burst suppression combined or separately (30).

In contrast, among an additional 45 patients in whom cerebral anoxia was the cause of RSE, induced burst suppression was associated with persistent seizure termination and survival in univariable analyses (30). However, the small sample size precluded adjusted analyses and limits the robustness of these observations. The ongoing RESTORE trial (NCT05851391), an open-label randomized study comparing burst suppression and seizure suppression strategies in adults with post–cardiac arrest RSE, is expected to provide further insight into this question. Importantly, such findings should not be extrapolated to other RSE etiologies, as hypoxic–ischemic injury differs fundamentally in pathophysiology, prognostic framework, and therapeutic goals. In a subsequent study of 111 patients with RSE of non-anoxic etiology, we evaluated CIVAD dose escalation, defined as either an increase in dose or a switch to another agent (31). CIVAD escalation occurred in 57% of patients, most commonly involving midazolam (79%). Although patients undergoing dose escalation had significantly higher baseline morbidity and more frequent complications, no differences were observed in in-hospital mortality or return to premorbid neurological function compared with patients without escalation. This suggests that escalation of CIVAD therapy may mitigate the adverse prognostic impact of greater initial disease severity. While burst suppression was more frequently achieved in patients undergoing CIVAD escalation, this difference was not statistically significant (31).

In summary, the current evidence indicates that, for most patients with RSE, targeting EEG burst suppression does not provide additional benefit over seizure suppression alone. This has important clinical implications, as not all CIVADs are similarly effective to establish a burst suppression pattern, and attempting to do so may require higher doses, potentially increasing the risk of adverse effects.

## When should we start, and when should we stop? The question of CIVAD timing

Current guidelines advocate a stepwise, escalative treatment strategy for SE: benzodiazepines as first-line, followed by non-anesthetic ASMs, such as levetiracetam, valproic acid, or fosphenytoin as second-line treatment for established SE, with CIVAD reserved as third-line therapy for RSE (5–7). This approach is grounded in the conceptual SE framework defining two operational time points: *t1*, marking the transition to abnormally prolonged seizure activity, and *t2*, beyond which long-term consequences such as neuronal injury and functional impairment become increasingly likely. While these thresholds vary by SE type, they are best established for convulsive SE, with *t1* at approximately 5 min and *t2* at 30 min, and are less well defined for other SE subtypes (3).

Although *t1* and *t2* are operational rather than biologically fixed thresholds, they are informed by time-dependent changes in seizure biology (3). Early SE is associated with reduced GABAergic inhibition, partly due to synaptic GABA(A) receptor internalization and impaired chloride homeostasis, resulting in high benzodiazepine responsiveness. With ongoing seizure activity, seizure maintenance increasingly depends on non–GABA-dependent mechanisms, including enhanced glutamatergic transmission and activation of metabolic and inflammatory pathways, promoting a self-sustaining hyperexcitable network state (8). This conceptual framework underscores the importance of timely intervention and has motivated investigation into whether earlier initiation of anesthetic therapy may improve outcomes.

When should CIVAD be started? A retrospective single-center study of 77 patients with RSE compared outcomes between those receiving CIVAD within 48 h of RSE onset (69%) and those treated later (31%) (32). Early CIVAD initiation was associated with more favorable functional outcomes, shorter SE duration, and a lower likelihood of requiring burst suppression or thiopental. Subgroup analyses suggested that this benefit was largely confined to patients younger than 65 years, with a prior history of seizures and a Status Epilepticus Severity Score (STESS) <3 (32).

Similarly, a retrospective two-center study from Geneva and Basel involving 205 adult patients with SE compared CIVAD administered as second-line therapy (27%) with guideline-adherent third-line use (73%) (33). No difference in in-hospital mortality was observed. However, earlier anesthesia was associated with significantly shorter SE duration, reduced ICU and hospital length of stay, and no increase in complications. Importantly, early CIVAD was preferentially administered to younger patients with more severe SE presentation.

An expanded analysis of this cohort over a 7-year period provided a more granular assessment of timing (34). Among 246 SE patients treated with CIVAD, therapy was guideline-conforming in 21%, initiated earlier (first or second line) in 55%, and delayed in 24%. Each additional non-anesthetic ASM administered before anesthesia was independently associated with reduced odds of return to premorbid neurological function at discharge. Earlier CIVAD initiation was significantly linked to higher rates of return to premorbid function (53% vs. 36% for guideline-conforming or delayed initiation), fewer infections, and shorter SE duration (34). These associations remained significant after adjustment for potential confounders, including age, etiology, SE type and severity, and comorbidities.

Conversely, conflicting results emerged from other datasets. A *post hoc* analysis of the Rapid Anticonvulsant Medication Prior to Arrival Trial (RAMPART) showed that among 1,023 SE patients, 21% were intubated (with over 90% on site or within 6 h of hospital arrival). Any intubation was significantly associated with higher mortality (7% vs. 0.4% non-intubated patients) (35). Notably, intubation was prompted by ongoing seizure activity in only 48% of cases.

Similarly, an observational study from the multicenter Sustained Effort Network for treatment of Status Epilepticus (SENSE) registry compared 55 intubated patients receiving early first- or second-line CIVAD (regardless of indication SE treatment or airway protection) with 220 controls treated later (17%) or not at all (83%) with CIVAD (36). After propensity score matching, mortality, functional outcome, seizure cessation, and SE duration were comparable between patients receiving early CIVAD and controls.

When should CIVAD be stopped? No randomized controlled trials have yet addressed the optimal duration of anesthetic therapy for RSE. Current guidelines recommend maintaining CIVAD for 24–48 h after seizure control, a practice largely based on expert consensus (5–7, 17). However, multiple observational studies have demonstrated strong associations between prolonged sedation and complications, including infections, arterial hypotension, cardiac depression, propofol infusion syndrome, and gastrointestinal paralysis (10, 12–14). Additionally, prolonged sedation after resolution of SE was associated with delayed extubation and post-SE delirium (37, 38).

Despite these concerns, survey data indicate relative stability in clinical practice over time. In the aforementioned survey conducted in three European countries in 2003, 72% of clinicians reported maintaining CIVAD for 24–48 h, and 22% for less than 24 h (21). A 2025 follow-up survey showed similar preferences, with approximately 70% favoring 24–48 h, 5% less than 24 h, and nearly 20% adopting an individualized, not further specified, approach (22). Data from the global STEP Audit consortium further demonstrated substantial variability, with CIVAD duration of ≤24 h in 36% of RSE and super-RSE cases, and 1–7 days in 53% (39).

A multinational retrospective study of 386 patients with RSE found that longer CIVAD duration was associated with higher rates of breakthrough and withdrawal seizures, whereas longer continuous EEG monitoring was linked to shorter anesthetic exposure, especially in high-income countries (40).

A separate two-center retrospective study of 182 patients showed that seizure duration–adjusted CIVAD duration was independently associated with seizure recurrence, but not with mortality, complications, or functional outcome at discharge (41). Notably, baseline clinical characteristics did not differ between patients with and without seizure recurrence, suggesting a potential iatrogenic effect of prolonged anesthesia. The authors discussed that this could be possibly mediated by compensatory upregulation of excitatory glutamate receptors during prolonged anesthetic exposure, thereby increasing seizure risk during weaning (41).

In summary, the optimal timing for both initiation and discontinuation of CIVAD in (R)SE remains uncertain. Evidence supporting very early anesthetic use is conflicting; however, early administration is conceptually aligned with the evolving pathophysiology of SE, in which anesthetics may act during a period when GABAergic responses can still be effectively potentiated or activated. Notably, earlier CIVAD use could potentially reduce treatment duration by terminating SE before further reductions in GABAergic responsiveness and consolidation of seizure networks occur. With respect to treatment duration, prolonging CIVAD beyond seizure control appears to increase the risk of complications and seizure recurrence. Although the expert-recommended duration of 24–48 h lacks strong empirical support, it is widely adopted in clinical practice.

## Whom should we treat? The question of patient selection for CIVAD

Convulsive SE is an acute, life-threatening neurological emergency, and current guidelines primarily address its management, reflecting the strongest available evidence levels (5–7). Recommendations for CIVAD use are therefore largely derived from studies of convulsive SE and extrapolated to other SE subtypes, despite ongoing debate regarding the risk–benefit balance of anesthetic therapy in nonconvulsive forms of RSE.

A retrospective single-center study investigating factors associated with CIVAD use as third-line therapy found that higher ictal burden, lower Glasgow Coma Scale score at the time of escalation, absence of a prior epilepsy diagnosis, and greater comorbidity burden were independently associated with CIVAD initiation (42).

Expert consensus supports aggressive treatment of subtle SE, a late manifestation of prolonged convulsive SE characterized by minimal or absent motor activity with ongoing ictal EEG patterns (electromotor dissociation), as subtle SE is generally regarded as a continuation of convulsive SE rather than a distinct entity, justifying similar therapeutic intensity (43, 44).

In contrast, evidence guiding CIVAD use in NCSE is limited and heterogeneous. NCSE encompasses a broad clinical spectrum, ranging from mild impairment of consciousness in typical absence SE to profound coma. Prognosis correlates strongly with the extent of the impairment of consciousnessrather than with EEG features alon (45). Typical absence SE lacks evidence of neuronal injury comparable to convulsive SE. Pathological studies and animal experiments examining the neurotoxic sequelae of NCSE are rare (43). At the other end of the spectrum, refractory NCSE with coma has consistently been associated with poor outcomes (1, 45, 46). A central unresolved issue is whether coma in NCSE reflects the epileptic activity itself or the underlying precipitating pathology, complicating decisions regarding escalation to CIVAD (47, 48).

Reports of CIVAD use in NCSE without coma are sparse. Typical absence SE in patients with idiopathic generalized epilepsy syndromes is almost universally non-refractory, responds well to standard ASM, and carries an excellent prognosis without anesthetic therapy (43, 49).

Focal NCSE with impaired consciousness (previously also described as complex-partial or dyscognitive SE) has historically shown favorable responses to ASMs, with outcomes largely determined by etiology (50–53). In a more recent single-center retrospective study of 77 patients with focal NCSE with impaired consciousness, prolonged SE duration (>100 h) and etiology were the primary drivers of poor 4-year long-term outcome; only one patient received CIVAD (54). Similarly, a study examined 45 patients older than 60 years. Two-thirds experienced clinical improvement within 24 h, which was associated with survival (55). Thirty-day mortality occurred in 22%, with one-third attributed to SE-related complications and the remainder to comorbidities or unrelated causes.

Repeated or persistent focal aware motor SE, also termed epilepsia partialis continua (EPC), is frequently refractory to first-line ASMs but is rarely treated with CIVAD. A systematic review of 51 adult patients identified only one case receiving anesthetic therapy (56). A multinational case series of 65 pediatric and adult patients with EPC, not included in the systematic review, described two cases with failed thiopental treatment (57). In the SENSE registry, 18% of 240 patients with focal RSE with or without impaired consciousness or refractory absence SE were intubated for CIVAD, although reporting granularity is limited (58).

Taken together, current evidence indicates that CIVAD has a negligible role in NCSE without coma and in focal-motor SE, where outcomes are largely driven by etiology and comorbidities rather than seizure persistence alone.

In contrast, the majority of NCSE encountered in critically ill patients consists of NCSE with coma or subtle SE, conditions associated with substantial morbidity and mortality that are difficult to disentangle from the underlying systemic or neurological injury (48, 59). Evidence supporting aggressive treatment comes from prospective data showing that subtle SE (defined as coma with ictal EEG discharges, with or without subtle motor movements) responded poorly to first-line ASM in only 15% compared to 55% in convulsive SE, providing a rationale for further escalation (44). However, interpretation across studies is complicated by the frequent inclusion of mixed SE populations and the absence of analyses focused exclusively on NCSE subtypes (60). Moreover, while the underlying cause of SE appears to influence the likelihood of neurological recovery, it does not independently predict short-term mortality once confounders and decisions regarding withdrawal of life-sustaining therapy are taken into account. In contrast, NCSE accompanied by coma is most strongly linked to unfavorable outcomes (61). This further complicates assessment of the independent impact of CIVAD or similar therapies, as observed outcomes likely reflect the combined effects of disease severity, underlying pathology, and treatment strategies rather than the effect of any single intervention.

Given these uncertainties, current expert guidance emphasizes individualized treatment decisions. Factors such as comorbidity burden, SE severity, etiology, and trajectory and potential of neurological recovery should guide CIVAD use without exerting unjustified nihilism (60, 62). Initial optimization of broad-spectrum ASMs may be preferable before escalation to CIVAD therapy with attention to minimizing duration once seizure control is achieved (59).

## Discussion

This narrative review examined the evidence supporting CIVAD use in the management of SE, focusing on three central clinical questions: the optimal titration target, the timing of initiation and discontinuation, and appropriate patient selection. Several important limitations of the available literature must be acknowledged. In addition, as a narrative scoping review, this work did not follow a formal PRISMA-guided systematic review methodology. Most evidence derives from observational studies and is therefore prone to selection bias and confounding by indication, particularly in the absence of randomized controlled trials in RSE. Despite statistical adjustment, residual confounding remains a major concern. In addition, substantial heterogeneity in SE definitions, patient populations, CIVAD protocols, and outcome measures limits direct comparison across studies. The predominance of data from tertiary referral centers in high-income countries further constrains generalizability.

This review did not focus on direct comparisons between individual CIVAD agents. Overall, the current literature does not demonstrate a superiority of propofol over midazolam or vice versa (29, 63, 64). Barbiturates appear to achieve higher rates of seizure control, but at the cost of significantly increased complications. Ketamine represents a promising adjunctive agent, particularly in prolonged or super-RSE, but comparative data remain sparse and insufficient to support firm conclusions and were therefore not emphasized in this review (43). Notably, ketamine has predominantly been used late in the treatment course of RSE and super-RSE (65, 66). Given evidence for early upregulation of NMDA receptors during SE, it is tempting to consider ketamine use earlier in the pharmacological treatment sequence of established SE (67). To date, this approach has been explored in only a limited number of studies, although the available results are promising (68). The ongoing Ketamine Add-on Therapy for Established Status Epilepticus Treatment Trial (KESETT; NCT06907173) is expected to provide further insight into this strategy.

Beyond considerations of timing, ketamine’s NMDA receptor antagonism offers mechanistic complementarity to GABAergic anesthetics, supporting the concept of mechanism-informed combination therapy targeting both impaired inhibition and excessive excitation. Emerging interest in dual glutamatergic strategies, such as combining ketamine with AMPA receptor antagonists (e.g., perampanel), reflects a shift toward pathophysiology-driven rather than purely escalation-based treatment approaches, although robust comparative data remain lacking (69).

With respect to titration goals, current evidence does not support burst suppression as superior to seizure cessation in terms of mortality or functional outcome (18, 19, 30). Moreover, the practical challenges of achieving and sustaining burst suppression over prolonged periods render seizure cessation a more pragmatic and widely applicable target for most patients with RSE (24). Important uncertainties persist, however, including whether specific subgroups, such as patients with super-RSE or post-anoxic RSE, may benefit from deeper levels of anesthesia (43), and whether qualitative or quantitative features within burst suppression (e.g., suppression ratio variability or highly epileptiform bursts) have therapeutic or prognostic relevance (70–72).

The timing of CIVAD initiation remains uncertain. Studies examining guideline-deviating first- or second-line anesthetic use have yielded inconsistent results (33, 34, 36), and broader evidence on the impact of non-adherence to guideline-recommended treatment sequences is similarly conflicting (73–75). Nonetheless, early intubation was not associated with a higher risk of adverse events (34, 36), and delayed initiation of CIVAD in established RSE is consistently associated with worse outcomes, underscoring the importance of timely escalation once refractoriness is established (1, 4, 14).

Evidence guiding the optimal duration of CIVAD therapy is limited. Although current guidelines recommend maintaining anesthesia for 24–48 h after seizure control, this recommendation rests largely on expert consensus rather than robust empirical data and has been perpetuated through clinical practice (5–7, 17, 21, 22). However, shorter windows should always be considered. Continuous EEG monitoring is essential during both maintenance and weaning phases (76). Emerging approaches, including advanced EEG connectivity analyses and novel prognostic scores aimed at predicting successful weaning, are promising but remain investigational and lack external validation (77, 78).

Patient selection likely represents the most critical aspect of CIVAD use. Poor functional outcomes are strongly associated with advanced age, comorbidity burden, etiology, and comatose presentations of NCSE (1). However, the observational nature of available data precludes determination of whether CIVAD modifies outcomes in these high-risk populations or merely reflects disease severity. What is clear is that CIVAD carries substantial risks, including prolonged mechanical ventilation, infections, and hemodynamic instability (10–13). Current data provide little support for its use in refractory absence status, focal motor SE (including EPC), or in NCSE without impaired consciousness, where the balance of risk and benefit appears unfavorable (54–57).

**Figure 1:**
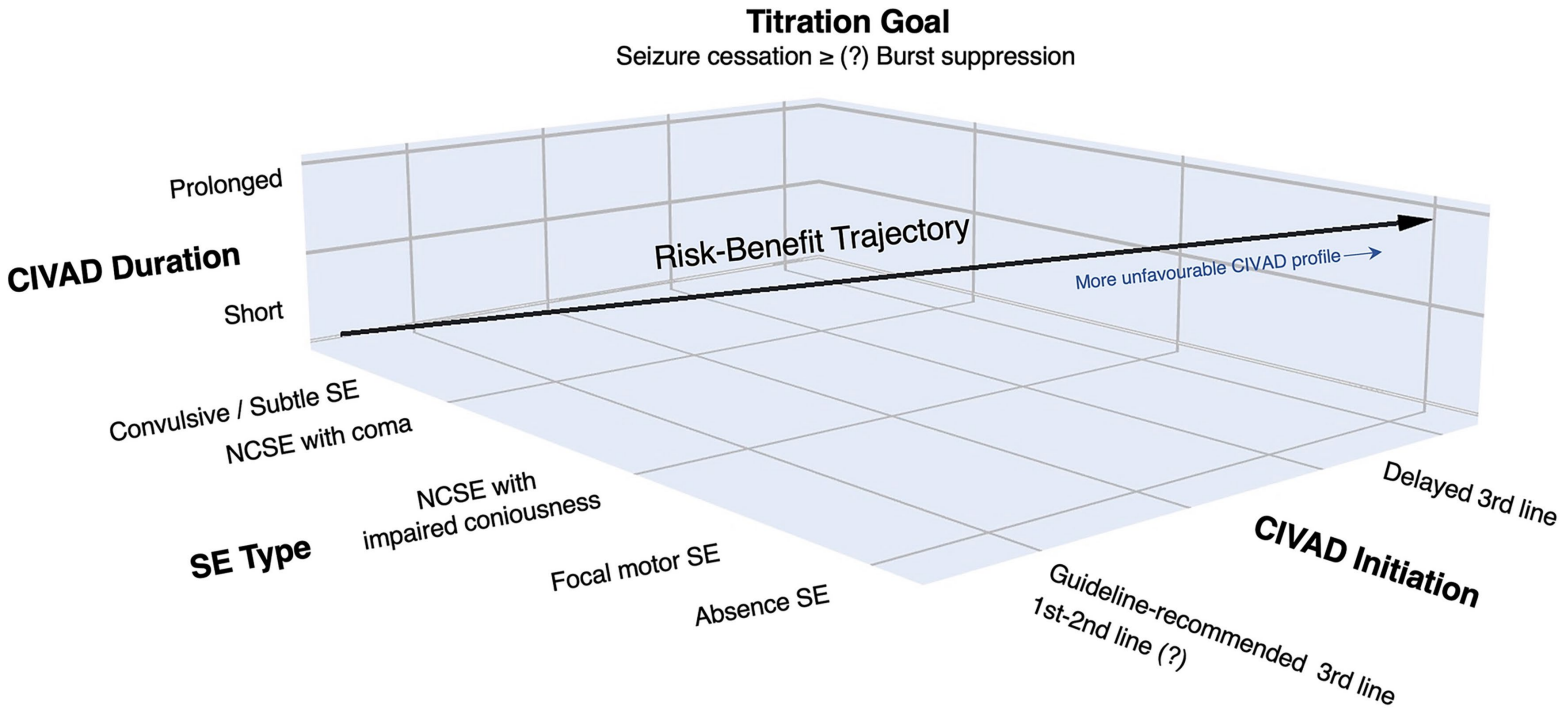
Conceptual framework of the risk–benefit balance of CIVAD therapy in SE. The schematic integrates status epilepticus type, timing, and duration of CIVAD therapy, and titration goal to illustrate how the balance between potential benefit and treatment-related harm may shift across clinical contexts. The diagonal trajectory reflects a transition from more favorable to increasingly unfavorable trade-offs with delayed CIVAD initiation, prolonged exposure, and use in less immediately life-threatening SE phenotypes. Question marks indicate areas of uncertainty, including CIVAD use prior to guideline-recommended escalation and the optimal titration target, where burst suppression may not confer additional benefit over seizure cessation alone. CIVAD, continuous intravenous anesthetic drug; NCSE, nonconvulsive status epilepticus; SE, status epilepticus.

Figure 1 synthesizes the key considerations discussed in this review, SE phenotype, timing of CIVAD initiation and duration, and titration goal, by framing CIVAD therapy as a dynamic risk–benefit trade-off, underscoring that maximal anesthetic intensity is not uniformly advantageous and should be tailored to the individual clinical context.

Collectively, persistent gaps in evidence, including uncertainty about patient selection, titration goals, and the timing and duration of CIVAD therapy, highlight the need for a paradigm shift in RSE management. Rather than relying on standardized protocols derived primarily from expert opinion, future efforts should prioritize personalized treatment strategies incorporating patient-specific characteristics, pathomechanisms, and real-time physiological data (79). Large prospective registries with standardized data collection may help define clinically meaningful subgroups and inform hypothesis generation (80). Ultimately, pragmatic randomized trials comparing tailored treatment strategies within defined RSE populations are essential. Advances in EEG analytics, neuroimaging and other biomarkers may further refine prognostication and therapeutic targeting. Until such evidence becomes available, clinicians must carefully balance the potential benefits of CIVAD against its risks, engage in shared decision-making when feasible, and recognize that maximal treatment intensity does not invariably align with patient-centered care.

## Conclusion

SE is a life-threatening neurological emergency in which the use of CIVAD requires careful balancing of potential life-saving effects against substantial treatment-related morbidity. The available evidence remains largely observational, but suggests that delayed CIVAD initiation once refractoriness is established is associated with poorer outcomes, supporting timely escalation in appropriately selected patients. Patient selection is therefore critical: CIVAD appears most justifiable in convulsive RSE or NCSE with coma, whereas in focal motor SE or NCSE with preserved consciousness, risks such as hemodynamic instability and prolonged mechanical ventilation may outweigh potential benefits.

From a practical standpoint, as a first titration goal, seizure cessation is a more achievable target than burst suppression, which is difficult to maintain and has not demonstrated superior functional or mortality outcomes. Although consensus guidelines suggest a 24–48 h maintenance period, clinicians should adopt a dynamic approach, considering shorter windows while utilizing continuous EEG as a mandatory tool for monitoring and weaning.

Advancing beyond expert consensus will require prospective comparative trials, standardized registries, and improved clinical tools, including advanced EEG analytics, biomarkers, and validated prognostic models. Until such data are available, clinicians must prioritize individualized, patient-centered decision-making, recognizing that a single approach does not fit all patients.
